# Dental patients’ views on oral health and hygiene: a questionnaire-based assessment in Bangladesh

**DOI:** 10.3205/dgkh000613

**Published:** 2026-01-09

**Authors:** Majedul Hoque, Md Aktaruzzaman, Md Nahid Hasan

**Affiliations:** 1 Department of Pharmacy, Jahangirnagar University, Dhaka, Bangladesh; 2 Department of Pharmacology and Experimental Therapeutics, University of Toledo, Ohio, USA

**Keywords:** oral health, oral hygiene, toothbrush, awareness

## Abstract

**Background:**

A major influence on quality of life is caused by the general public's inability to recognize poor oral health and untreated oral disorders, as well as the widespread acceptance of poor oral status as the standard. Because they are so common and often ignored, oral diseases pose an existential risk to public health. People's attitudes regarding oral health and illness, therefore, have a significant impact on dental health. This study aims to assess the patients' behavior, attitudes, and level of knowledge while visiting or seeking dental treatment at various dental care clinics in Kishoreganj, Bangladesh.

**Methods:**

A structured self-chosen questionnaire, that takes 5-10 minutes to complete, was posed to general patients visiting dental care. The individuals' responses were computed as percentages.

**Results:**

Among the 214 participants, 67% brush their teeth two times daily. About 72% patients change their toothbrush following 2 months of usage and 6% patients change it within 6 months. Among the patients 44% stated primary information source about dental health is their parents. Majority patients 84% know that oral health have influence over general well-being of our life and the practice of cleaning mouth and making gurgle after eating food is prevalent among participants by just below 50%.

**Conclusion:**

The study participants' attitudes, practice and awareness of their dental health are reasonable, but they fall short of what is needed. Consequently, it is essential to offer instruction and encouragement regarding oral health and associated challenges.

## Zusammenfassung

**Hintergrund:**

Ein wesentlicher Einflussfaktor auf die Lebensqualität ist die Unfähigkeit der Öffentlichkeit, schlechte Mundgesundheit und unbehandelte Munderkrankungen zu erkennen, sowie die weit verbreitete Akzeptanz eines schlechten Mundzustands als Norm. Da sie häufig vorkommen und oft ignoriert werden, stellen Munderkrankungen ein existenzielles Risiko für die öffentliche Gesundheit dar. Die Einstellung der Menschen zu Mundgesundheit und Munderkrankungen hat daher einen erheblichen Einfluss auf die Zahngesundheit. Die Studie zielt darauf ab, das Verhalten, die Einstellung und den Wissensstand von Patienten zu bewerten, die verschiedene Zahnkliniken in Kishoreganj, Bangladesch, aufsuchen oder sich dort zahnärztlich behandeln lassen.

**Methode:**

Ein strukturierter, selbst erstellter Fragebogen, für dessen Ausfüllen durchschnittlich 5-10 min benötigt werden, wurde Patienten, die eine Zahnarztpraxis aufsuchten, vorgelegt. Die Antworten wurden als Prozent berechnet.

**Ergebnisse:**

Von 214 Teilnehmenden putzen 67% ihre Zähne zweimal täglich. Etwa 72% der Patienten wechseln ihre Zahnbürste nach zwei Monaten Gebrauch und 5,6% der Patienten wechseln sie innerhalb von sechs Monaten. Unter den Patienten gab eine große Anzahl von Teilnehmern (44%) an, dass ihre Eltern die wichtigste Informationsquelle zum Thema Zahngesundheit sind. Die Mehrheit der Patienten (84%) weiß, dass die Mundgesundheit Einfluss auf das allgemeine Wohlbefinden hat, und knapp unter 50% der Teilnehmer putzen sich nach dem Essen die Zähne und gurgeln.

**Schlussfolgerung:**

Die Einstellung, die Gewohnheiten und das Bewusstsein der Studienteilnehmer hinsichtlich ihrer Zahngesundheit sind angemessen, reichen jedoch nicht aus. Daher ist es unerlässlich, Aufklärung und Unterstützung in Bezug auf die Mundgesundheit und die damit verbundenen Herausforderungen anzubieten.

## Introduction

A person's ability to eat, speaking, and interact with others without illness or discomfort is known as oral health, and it also contributes to overall health [1]. A significant and vital part of overall health is oral health. The relationship between oral disorders and systemic conditions such as diabetes, heart disease, stroke, metabolic syndrome, unfavorable pregnancy outcomes, digestive problems, obesity, etc., is well established [2]. The great frequency of oral disorders and their impact on an individual's quality of life make them a significant public health problem [3]. Additionally, there are oral pathologic situations where the repair and regeneration of oral tissues depend on proper oral hygiene. These ailments include periodontitis, gingivitis, and dental trauma such subluxation, oral cysts, and the aftermath of wisdom tooth extraction [4], [5], [6]. These oral disorders could be caused by a combination of developmental issues, non-compliance, poor oral hygiene, and genetic predispositions [7]. 

Several variables influence oral hygiene practices and the decision to seek dental care. Patients who get positive reinforcement and education are more likely to adhere to oral health care regimens. One of the causes of noncompliance with oral hygiene procedures is a lack of knowledge. Additionally, attitudes and beliefs about oral health have a big impact on oral health behavior [8]. Both the patient and the dentist must work together to maintain a healthy oral profile. People's attitudes about their teeth are among the most significant determinants of a population's dental health [9]. 

Oral health affects a person's social, physiological, and physical health. Chronic diseases that negatively affect oral and general health, such as diabetes, obesity, and dental caries, are on the rise in developing countries [10], [11]. Most people have a tendency to ignore oral problems that could worsen and affect their overall health. Due to people's ignorance of this reality, millions of individuals endure unbearable toothaches and a low quality of life [12]. Although there appears to be only a weak correlation between knowledge and behavior in cross-sectional studies, research has indicated that knowledge is associated with improved oral health, thus explaining why oral health knowledge is regarded as a necessary precondition for health-related behavior [13], [14]. So, it is necessary to assess the patients' behavior, attitudes, compliance to oral standard and level of knowledge. 

## Methods and material

### Study area

This study was conducted in Kishoreganj district in April 2025 among random patients visiting or seeking medical advice at various dental care clinics in Kishoreganj, Bangladesh. 

### Ethical clearance

After receiving formal informed consent from all the patients, all participants were asked orally to answer the questionnaires after receiving explanations on the purpose of the study.

### Exclusion criteria

This comprised of participants who were not willing to partake in the survey. Further, Dentists, dental students, dental assistants, and dental nurses were excluded. 

### Questionnaire

Participants were asked questions in their native tongue and translated into English. The first section of the questionnaire collected demographic data from respondents, including age, sex, gender, and occupation. After that, questionnaire used for collecting information about oral health from the study participants are

- How many times do you brush a day?
- How frequently change your tooth brush?
- How many minutes you take to brush your teeth?
- Which type of dental aids do you use other than toothbrush?
- What is your main source of knowledge of dental health?
- Do you think dental health is important?
- Do you think oral health affects general health?
- How frequently do you visit the dentist?
- Do you know dental plaque is the main cause for tooth loss?
- Are you aware that sugar/sticky food can cause tooth decay?
- Are you aware that bleeding gums is a sign of poor gum health?
- Is it your first visit to dentist?
- Do you rinse your mouth after food consumption?

### Data analysis

The information was transferred to Microsoft Excel to summarize and for the analysis of data, SPSS software version 24 was used to statistically evaluate the results in terms of percentage. 

## Results

Among the 214 participants 47.2% are men and 52.8% are women. 33.6% of households are rural, while 66.4% are urban. Of these, 5.6% are illiterate, 42.5% have completed the tenth grade, 24.8% have completed the fifth, and 27.1% hold a higher degree (Table 1 (Tab. 1)). Their age range is 20 to 65 years.

After conducting the study, it was found that majority of the patients 66.82% brush their teeth two times daily, while 18.2% brushes teeth one time and nearly 15% patients do it three times a day. According to the commentary, about 72% patients change their toothbrush following 2 months of usage and 5.6% patients change it within 6 months. A sufficient number of patients 46.3% told that they brush their teeth for only 1 minute during every brushing. However, 37.4% stated 2 minutes is the ideal time of brushing and only 16.4% claimed it more than two times. On the other hand, 66.4% patients said they use mouthwash as a supplementary dental aid other than toothbrush but it was dental floss according to 33.6% patients. Among the participants 43.9% stated primary information source about dental health is their parents, 31.8% told it was dentist and dental clinic, nearly 17% told about Television and social media and only 7% ascribed their friends as a source of information (Table 2 (Tab. 2)). 

Most of the patients 96.7% believe that dental health is essential to lead a quality life and hardly 1% patients were against this belief, with 2.3% didn’t have any comment about it. Majority patients 84.1% know that oral health have influence over general well-being of our life, when, 15.9% participants were not aware of this issue and nobody opposed this issue. By considering frequency of dentist visiting, almost 87% patients said they only visit dentist if there is a dental problem occurs, and rest of the participants said they visit dental expert one or two time per year. While 54.2% patients know dental plaque is the principle cause of tooth loss, 45.8% respondents didn’t know so. It was a remarkable awareness among patients that 80% of them know about bad impact of sweet and sugary food on health that can accelerate dental decay unfortunately, 20% respondents were unaware of it. 93% participants know bleeding gum as a sign of poor gum health and rest few numbers are not. The practice of cleaning mouth and making gurgle after eating food is prevalent among participants by just below 50% but more than fifty percent participants didn’t possess this necessary healthy habit (Table 2 (Tab. 2)). 

## Discussion

The present study demonstrates that the general perspective on oral health is still ignored, and it finds a correlation between dental awareness and key demographic factors such as education, gender, and socioeconomic background. The awareness of rural and less educated population of Bangladesh is quite average. This inference is corroborated by observations made by Chandu et al. [15]. In the current study, it was found that 66.8% of participants brush twice a day which is closer to the findings by Oberai et al. [16], but significantly larger than 17% reported by Goryawala et al. [17]. Knowledge assessment is crucial for developing such oral health education. When someone is knowledgeable, they have all the information they need to comprehend oral disease, how it develops, and the precautions that should be taken. This information might cause the person to shift their perspective, which might then cause them to alter their day-to-day activities [18], [19]. 

The changing frequency of toothbrush by participants in the current study represents normal trends of two months which is close to study conducted by Oberai et al. and Casanova-Rosado et al. [16], [20]. Approximately 33% study patients used dental floss to clean their teeth that is lower compared to another study [21]. Even though over 87% of the patients in this sample understood the value of routine dental checkups when tooth issues arise, only 8.4% said they had seen a dentist in the previous six months. According to a research conducted in India, 35.1% of respondents had seen a dentist in the previous 12 months, and 67.8% of respondents understood the value of routine dental checkups. This conclusion is somewhat consistent with those findings [22]. 

In this survey, only 37.4% of the respondents said they brushed for two minutes. Conversely, 46.3% of them stated that they brush for just one minute. This demonstrates the necessity of raising knowledge of dental health and oral hygiene practices [23]. However, this study gives us information regarding the study participants’ educational background, which is tangentially related to their knowledge and health-related behaviors. The majority of people thought that dental health and general health were associated, according to the current study. Which indicates that, nowadays people at least have more knowledge about their health status compared to past. 

Understanding dental hygiene is crucial because it keeps our mouths healthy. The majority of participants in this survey learn from a variety of sources, including the media, friends, and family. While more than half of respondents cited other sources, 43.9% of patients in our study stated that their parents are their main source of information on oral health, suggesting that not all parents actively work to teach their children about oral health. These findings were in line with previous research, which further supports the idea that many parents are not teaching their children about dental health [24], [25]. Just 7% of the patients thought that bleeding gums was a normal aging-related event; they were unaware that it was actually caused by poor gum health. Although 93% of them acknowledged the connection between bleeding gums and poor gum health, the patients did not think it was necessary to see a dentist. This finding is quite different from another study conducted by Ganss et al. [10]. 

After eating, it’s customary in Bangladeshi culture to rinse your mouth with water. In addition to removing any leftover food particles, it also removes some bacteria and the acids they create from the oral cavity. According to Winner et al. [26], 67% of parents have observed that they force their children to spit and swish with water after each meal [26]. This study showed, just 47.7% of patients practiced mouth washing after meal, which percentage is comparatively low. 

A comprehensive strategy that incorporates personal accountability, medical treatment, and public health campaigns is needed to maintain proper dental hygiene and wellness. The cornerstones of personal preventative practices continue to be daily interdental cleaning, using fluoride toothpaste on a regular basis, and using antimicrobial mouth rinses when necessary. The risk of dental caries and periodontal disease can be considerably decreased by eating a balanced diet low in refined sugars and drinking enough water. In order to facilitate the early detection and treatment of oral disorders, it is recommended that regular dental examinations and professional cleanings be promoted. Promoting population-level oral health equity requires public health initiatives like accessible preventative care, school-based oral health education, and community engagement.

## Limitations of study

First, a questionnaire was used to gather the data, and it's possible that participants overstated good habits and understated bad ones. Second, one should take into account recollection bias, which most likely arises in connection with previous dental appointments and dietary habits.

## Conclusion

The awareness and attitude of the study participants toward their dental health are quite fair but below the required level. Therefore, it is necessary to provide education and motivation on oral health and related difficulties. It is necessary to extend this approach outside the clinics by implementing different outreach initiatives in places that are more difficult for oral health facilities to reach. Furthermore, the media has the power to inform the public and promote routine dental checkups. To sum up, it is our responsibility to inform and motivate this population of individuals who do not have a formal education in order to move closer to creating a healthy environment.

## Notes

### Authors’ ORCIDs

- Hoque M: https://orcid.org/0009-0001-9044-411X
- Aktaruzzaman M: https://orcid.org/0009-0009-8784-7880
- Nahid Hasan MN: https://orcid.org/0000-0002-6409-3272

### Ethical approval

Selected outdoor and ambulatory patients provided informed consent orally, no other permission needed as it is not conducted in a particular institutions and organizations. 

### Funding

None 

### Competing interests

The authors declare that they have no competing interests.

## Figures and Tables

**Table 1:**
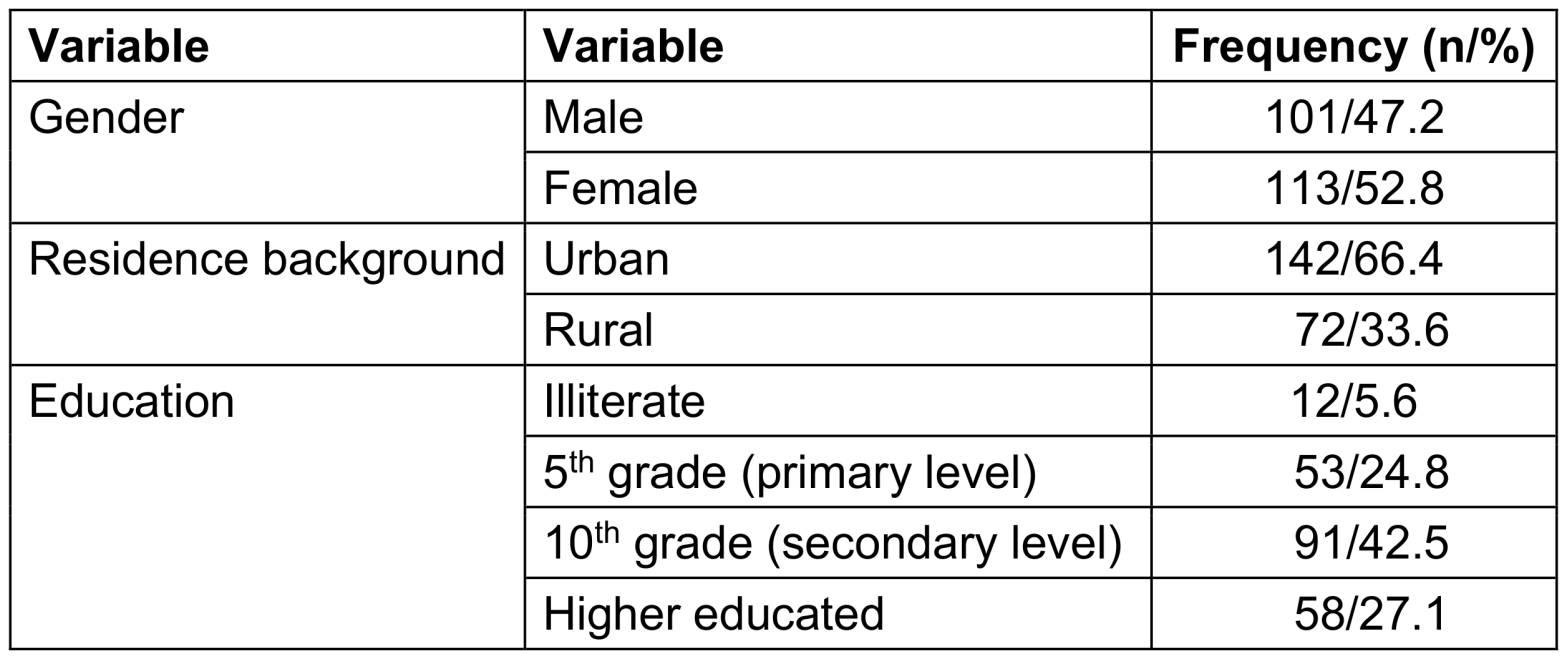
Socio-demographic profile of participants

**Table 2:**
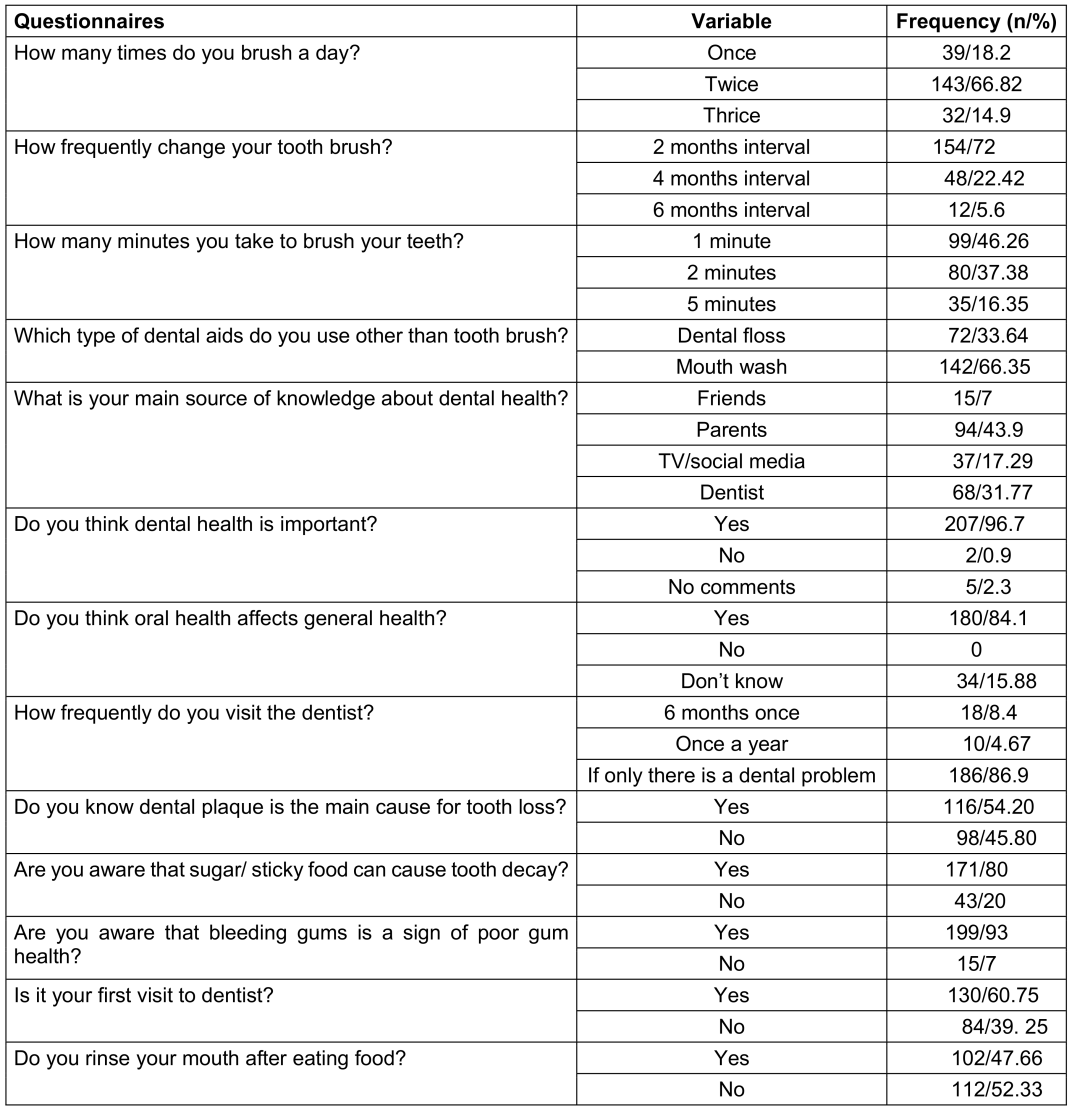
Habits and knowledge of participants about oral health and oral hygiene

## References

[1] Singh A, Gambhir RS, Singh S, Kapoor V, Singh J (2014). Oral health: How much do you know? - A study on knowledge, attitude and practices of patients visiting a North Indian dental school. Eur J Dent.

[2] Tavares M, Lindefjeld Calabi KA, San Martin L (2014). Systemic diseases and oral health. Dent Clin North Am.

[3] Butt AM, Ahmed B, Parveen N, Yazdanie N (2009). Oral health related quality of life in complete dentures. Pak Oral Dent J.

[4] Zadik Y (2008). Algorithm of first-aid management of dental trauma for medics and corpsmen. Dent Traumatol.

[5] Flores MT, Andersson L, Andreasen JO, Bakland LK, Malmgren B, Barnett F, Bourguignon C, DiAngelis A, Hicks L, Sigurdsson A, Trope M, Tsukiboshi M, von Arx T, International Association of Dental Traumatology (2007). Guidelines for the management of traumatic dental injuries. I. Fractures and luxations of permanent teeth. Dent Traumatol.

[6] Bautista CRG, Milhan NVM, Ankha MDVEA, do Prado RF, Cavalcante ASR, Lopes SLPC, Anbinder AL (2019). Bilateral mandibular buccal bifurcation cyst: a case report emphasizing the role of imaging examination in the diagnosis. Autops Case Rep.

[7] Tash RH, O'Shea RM, Cohen LK (1969). Testing a preventive-symptomatic theory of dental health behavior. Am J Public Health Nations Health.

[8] Chander Shekar BR, Reddy C, Manjunath BC, Suma S (2011). Dental health awareness, attitude, oral health-related habits, and behaviors in relation to socio-economic factors among the municipal employees of Mysore city. Ann Trop Med Public Health.

[9] Al-Wesabi AA, Abdelgawad F, Sasahara H, El Motayam K (2019). Oral health knowledge, attitude and behaviour of dental students in a private university. BDJ Open.

[10] Ganss C, Schlueter N, Preiss S, Klimek J (2009). Tooth brushing habits in uninstructed adults--frequency, technique, duration and force. Clin Oral Investig.

[11] Kwan SY, Holmes MA (1999). An exploration of oral health beliefs and attitudes of Chinese in West Yorkshire: a qualitative investigation. Health Educ Res.

[12] Cinthya R, Mohan R, Vijayakumar P, Dayanidhi R, Ramakrishnan H (2024). Assessment of oral health awareness among the individuals in Chengalpttu District: a questionnaire based study. Asian J Dent Health Sci.

[13] Kamble VS, Biradar SM, Takpere A, Reddy S (2017). Evaluation of oral hygiene awareness and practices among medical students. Int J Community Med Public Health.

[14] Kapoor D, Gill S, Singh A, Kaur I, Kapoor P (2014). Oral hygiene awareness and practice amongst patients visiting the Department of Periodontology at a Dental College and Hospital in North India. Indian J Dent.

[15] Chandu VC, Pachava S, Viswanath V, Khanna A, Kaur R (Int). Strategies for improving accessibility to oral health care services in Rural India: an insight.

[16] Oberoi SS, Mohanty V, Mahajan A, Oberoi A (2014). Evaluating awareness regarding oral hygiene practices and exploring gender differences among patients attending for oral prophylaxis. J Indian Soc Periodontol.

[17] Goryawala SN, Chavda P, Udhani S, Pathak NV, Pathak S, Ojha R (2016). A survey on oral hygiene methods practiced by patients attending Dentistry Department at a Tertiary Care Hospital from Central Gujarat. J Int Soc Prev Community Dent.

[18] Al-Omiri MK, Al-Wahadni AM, Saeed KN (2006). Oral health attitudes, knowledge, and behavior among school children in North Jordan. J Dent Educ.

[19] Smyth E, Caamano F, Fernández-Riveiro P (2007). Oral health knowledge, attitudes and practice in 12-year-old schoolchildren. Med Oral Patol Oral Cir Bucal.

[20] Casanova-Rosado JF, Vallejos-Sánchez AA, Minaya-Sánchez M, Medina-Solís CE, De La Rosa-Santillana R, Márquez-Corona Mde L, Maupomé G (2013). Frequency of tooth brushing and associated factors in Mexican schoolchildren six to nine years of age. West Indian Med J.

[21] Sharda AJ, Shetty S, Ramesh N, Sharda J, Bhat N, Asawa K (2011). Oral health awareness and attitude among 12-13 year old school children in Udaipur, India. Int J Dent Clin.

[22] Harikiran AG, Pallavi SK, Hariprakash S, Ashutosh, Nagesh KS (2008). Oral health-related KAP among 11- to 12-year-old school children in a government-aided missionary school of Bangalore city. Indian J Dent Res.

[23] Han K, Park JB (2017). Association between oral health behavior and periodontal disease among Korean adults: The Korea national health and nutrition examination survey. Medicine (Baltimore).

[24] Lertpimonchai A, Rattanasiri S, Arj-Ong Vallibhakara S, Attia J, Thakkinstian A (2017). The association between oral hygiene and periodontitis: a systematic review and meta-analysis. Int Dent J.

[25] Macek MD, Atchison KA, Chen H, Wells W, Haynes D, Parker RM, Azzo S (2017). Oral health conceptual knowledge and its relationships with oral health outcomes: Findings from a Multi-site Health Literacy Study. Community Dent Oral Epidemiol.

[26] Winnier JJ, Parmar A, Mehta S, Bambal K, Bhatia R (2015). oral hygiene maintenance in children-a survey of parental awareness. Int J Oral Health Med Restaurant.

